# Dietary Melatonin Supplementation Improved Intestinal Health and Immune Function of Pacific White Shrimp (*Litopenaeus vannamei*) Under High Alkali Stress

**DOI:** 10.3390/life15050772

**Published:** 2025-05-12

**Authors:** Yiming Li, Yucong Ye, Haojuan Yuan, Zongli Yao, Yan Li, Zhen Sun, Yuxing Wei, Yunlong Zhao, Qifang Lai

**Affiliations:** 1 East China Sea Fisheries Research Institute, Chinese Academy of Fishery Sciences, Shanghai 200090, China; liym@ecsf.ac.cn (Y.L.); yaozl@ecsf.ac.cn (Z.Y.); ly508109@163.com (Y.L.); sunzhen@ecsf.ac.cn (Z.S.); weiyx@ecsf.ac.cn (Y.W.); 2 Key Laboratory of Inland Saline-Alkaline Aquaculture, Ministry of Agriculture and Rural Affairs, Shanghai 200090, China; 3 School of Life Science, East China Normal University, Shanghai 200241, China; ycy_666@yeah.net (Y.Y.); 17634990808@163.com (H.Y.)

**Keywords:** melatonin, immune response, intestinal health, high alkali stress

## Abstract

The intestinal tract serves as a critical immune regulator in aquatic species, maintaining homeostasis and environmental stress resistance. This study evaluates the protective effects of melatonin (MT) on *Litopenaeus vannamei* (*L. vannamei*) under acute alkaline stress through a comprehensive analysis of intestinal morphology, antioxidant responses, apoptosis regulation, and microbial community dynamics. A total of six groups of melatonin treatment groups were designed. After another 2 months of breeding, a 96 h acute alkalinity stress experiment was conducted. Experimental supplementation revealed dose-dependent outcomes: 82.7 mg/kg MT significantly improved survival rates without affecting growth parameters, while higher concentrations (329.2 mg/kg) induced elevated apoptosis (*p* < 0.05). Histological examination demonstrated mitigated intestinal structural damage in MT-treated groups compared to non-supplemented controls under alkaline stress. Antioxidant capacity initially increased and then stabilized at optimal MT doses (82.7–165.1 mg/kg), accompanied by enhanced immune marker expression (*p* < 0.05). Microbial profiling indicated MT-mediated enrichment of commensal bacteria associated with polysaccharide metabolism, energy utilization, and intestinal immunity. This study establishes that melatonin exerts dose-dependent protection in *L. vannamei* under alkaline stress, balancing antioxidant enhancement, apoptosis modulation, and microbiome regulation to fortify intestinal health, with 82.7–165.1 mg/kg identified as the optimal therapeutic range for mitigating environmental stress without compromising physiological homeostasis. The results of this study establish an empirical framework for optimizing MT application in crustacean aquaculture, particularly highlighting its role in maintaining intestinal barrier integrity and microbial homeostasis under alkaline environmental challenges.

## 1. Introduction

Melatonin (N-acetyl-5-methoxytryptamine, MT) is an indoleamine derived primarily from the essential amino acid tryptophan and is associated with skin pigment accumulation in most animals [1]. Melatonin regulates a variety of physiological functions, including the regulation of sleep–wake rhythms, the reproductive system, and hormone balance [1,2]. In addition, melatonin enhances the innate immunity, growth performance, and feed utilization of aquatic animals [3]. It is distributed in various tissues and organs of crustaceans, such as hemolymph, eyestalks, and the gut [4,5,6], and modulates the growth and molting of crustaceans, and it can enhance their resistance to adverse environmental factors [5,6,7,8]. Consequently, the use of melatonin in aquaculture feeds, including aquacultural feed, has gradually increased.

Light exposure regulates melatonin synthesis through a series of enzymatic reactions in organisms [9]. In standard aquaculture conditions, the critical role of photic regulation in melatonin biosynthesis is frequently overlooked, which may lead to alterations in crustacean growth patterns and physiological homeostasis [10]. Consequently, exogenous melatonin supplementation becomes imperative to optimize growth performance and enhance stress adaptation mechanisms in cultured crustaceans. Exogenous melatonin supplementation can reduce oxidative stress by inhibiting the formation of hydroxyl radicals and inhibiting the inflammation and apoptosis of damaged cells [11]. Melatonin is a free radical scavenger and metal-chelating agent that protects cellular lipids and proteins from being oxidized [12]. It can also indirectly enhance the function of antioxidant enzymes, such as glutathione, glutathione peroxidase, and superoxide dismutase [13]. Dietary supplementation of 75–81 mg/kg melatonin improved the survival and antioxidant and nonspecific immunity of the crayfish, *Cherax destructor* [14]. Li, et al. [15] found that 165.1 mg/kg melatonin supplementation in the diet of red swamp crayfish (*Procambarus clarkii*) significantly improved growth performance and antioxidant immunity, while Ref. [16] reported that dietary supplementation with melatonin improved the antioxidant, immune, and antibacterial defenses of Chinese mitten crab, *Eriocheir sinensis*.

A high carbonate content will lead to alkaline water, which affects the acid-base level in aquatic organisms, impacting enzyme activity and biochemical reactions and, thus, the growth and development, physiological function, cell metabolism, and even death of aquatic animals [17,18]. A series of experiments on the stress response of crustaceans in alkaline environments revealed that alkalinity stress causes tissue damage to the hepatopancreas, gills, muscles, and intestines [19], thus affecting the physiological state of organisms. Under acute stress of high alkalinity, significant damage occurred in the gills and hepatopancreas tissues of *L*. *vannamei*, accompanied by apoptosis [20,21]. In addition, high alkaline stress can also damage the immune function of aquatic organisms. For example, *E. sinensis* experienced increased oxidative stress under carbonate alkalinity, which impaired its immune function [22]. Therefore, feed nutrition fortification can be used as a quick effective way to improve the stress resistance of aquatic organisms. Previous work showed that the addition of different substances to the feed can mitigate the effects of high alkalinity, such as the addition of lipids and astaxanthin [23,24]. However, the effects of adding melatonin to feed on gut health and immunity have not yet been studied.

The intestinal tract is an important immune organ of crustaceans and is involved in the regulation of various physiological activities [25,26]. With its extensive surface area, the intestine serves as a site for the absorption and digestion of nutrients, while also acting as a robust barrier against pathogens [27]. The intestinal microflora not only participates in digestion and absorption but also has an important role in maintaining host health [28,29]. MT also has an important role in gut health, alleviating a variety of gastrointestinal diseases and intestinal damage [30]. Melatonin regulates intestinal movement in goldfish by attenuating neurotransmitter-mediated contractions [31] and can alleviate imidacloprid-induced pyroptosis and ferroptosis in the gut of the common carp through the PGN/TLR2/P38MAPK pathway [32]. Song, et al. [33] found that dietary supplementation with melatonin improved the antibacterial ability and intestinal microbial diversity of glyphosate-exposed *E. sinensis*. The gut is one of the main sources of melatonin. Melatonin has been shown to have beneficial effects on gut barrier function and the balance of microbial populations [34]. Therefore, by studying the effect of melatonin on the gut of crustaceans, we can better understand the mechanism of melatonin on their physiological regulation.

As a crucial commercial species in global aquaculture, *L*. *vannamei* serves as an essential source of high-quality animal protein for human nutrition [35]. Its widespread popularity can be attributed to its delicious meat, fast growth rate, high yield, and good economic benefits [35]. Nevertheless, emerging evidence indicates that alkalinity stress has become an urgent challenge in modern aquaculture systems, exacerbated by intensive farming practices and cumulative environmental pressures [36]. Indirectly, various chemicals and antibiotics are overused to ensure shrimp growth [37]. To address this critical issue, developing novel feed additives capable of ameliorating alkalinity stress while enhancing innate immunity represents a priority for sustainable shrimp farming. This investigation, therefore, systematically evaluates the therapeutic potential of dietary melatonin supplementation in improving immune competence and intestinal barrier function of *L. vannamei* under alkalinity stress conditions. Our findings establish a scientific foundation for employing melatonin as an eco-friendly immunomodulator, offering practical solutions to mitigate alkalinity-related challenges in intensive aquaculture operations.

## 2. Materials and Methods

### 2.1. Diet Preparation

White crystalline MT powder (with a purity of at least 99.0%) was obtained from China National Pharmaceutical Group Chemical Reagent Co. (Shanghai, China). For the experiment, a basal feed formulation (Table 1) was used as a base, supplemented with different levels of MT. To prepare the experimental diets, solutions of MT were dissolved in 1 mL of ethanol and sprayed onto the basal feed at various concentrations [38], followed by drying at 37 °C for 24 h. Six distinct experimental diets were formulated, containing varying concentrations of MT: 0 mg/kg (control), 20 mg/kg, 40 mg/kg, 80 mg/kg, 160 mg/kg, and 320 mg/kg MT. The true MT content in each feed was confirmed through ultra-high-performance liquid chromatography with ultraviolet detection (UPLC-UV). All of the diets were kept at −20 °C until required for use.

### 2.2. Experimental Shrimp and Feeding Management

The *L. vannamei* utilized in this study were sourced from Jinshan Aquafarm (Shanghai, China). Before the start of the study, the shrimp underwent a 7-day acclimatization period in 300 L tanks. For the experiment, 720 healthy shrimp, with a mean weight of 0.322 ± 0.005 g and a mean length of 2.423 ± 0.012 cm, were randomly allocated across 18 100 L tanks. Each experimental treatment was replicated three times, with 40 shrimp per tank.

The feeding regimen lasted for 2 months; the shrimp were fed three times daily (08:00 h, 14:00 h, and 20:00 h) with a quantity of food equivalent to 5% of their body weight. Water quality management included partial water changes every second day, exchanging half of the water volume, alongside the daily removal of deceased shrimp, leftover feed, and waste products. Water conditions were closely monitored and maintained as follows: dissolved oxygen levels at 6.7 ± 0.1 mg/L, temperature at 25 ± 2 °C, total ammonia nitrogen not exceeding 0.05 mg/L, pH at 8.1 ± 0.1, and salinity at 3 ± 0.5‰. Following the 2-month feeding period, ten shrimp from each treatment group were selected for the assessment of enzyme activity and gene expression levels. Concurrently, 60 shrimp of uniform size from each treatment group were selected to undergo an acute alkalinity stress test. All shrimp that received MT treatments were exposed to an alkaline environment of 350 mg/L, with each treatment group having three replicates, comprising 20 shrimp per replicate. The acute alkalinity stress lasted for 96 h, after which the survival rate was determined, and the surviving shrimp were sampled for further analysis.

### 2.3. Paraffin-Embedded Tissue Sections of Intestinal

After alkalinity stress, intestinal tissues of all treated groups were fixed in a 4% paraformaldehyde solution for more than 24 h, dehydrated through graded ethanol solutions (80%, 90%, 95%, and 100% for 45, 45, 30, and 30 min, respectively), embedded in paraffin, and sectioned into ultrathin slices. The sections were stained with hematoxylin and eosin (H&E) and examined under a bright-field microscope (ECLIPSE 90i; Nikon Corporation, Shinagawa-ku, Tokyo, Japan). Images were analyzed using Image-Pro Plus version 6.0 software (Media Cybernetics, Rockville, MD, USA), and morphometric measurements, including fold height (FH), fold width (FW), muscle layer thickness (MLT), and muscle thickness (mt), were quantified using Elements version 4.60 software (Nikon Instruments Inc., Minato-ku, Tokyo, Japan).

For apoptosis detection, tissue sections were digested with 100 μL of proteinase K, dried at room temperature for 5 min, and labeled with a 1× TdT labeling solution containing 2 μL of enzyme at 37 °C for 1 h in the dark. Following three gentle washes with PBS, nuclei were stained with DAPI for 3 min, and the sections were mounted with an anti-hardening medium before observation under a fluorescence microscope (Eclipse CI-L; Nikon Instruments Inc., Minato-ku, Tokyo, Japan).

### 2.4. Determination of Oxidative Stress-Related Indicators in Intestinal

Four shrimp were randomly selected from each group before and after alkalinity stress, and their intestinal tissues were collected and processed for analysis. The tissue samples were homogenized in a pre-prepared buffer solution at a ratio of 1:9 (tissue to buffer) and then centrifuged at 2500 rpm for 10 min at 4 °C. Following centrifugation, the supernatant was collected to assess various oxidative stress markers, including antioxidant enzymes (superoxide dismutase (SOD), catalase (CAT)), lipid peroxidation (LPO), malondialdehyde (MDA), acid phosphatase (ACP), alkaline phosphatase (AKP), glutamate oxaloacetate transaminase (GOT), and glutamate pyruvate transaminase (GPT). The enzymatic activities were measured using commercial kits supplied by Nanjing Jiancheng Bioengineering Institute, following the manufacturer’s protocols. SOD activity was defined as the quantity of tissue extract required to induce a 50% inhibition of the xanthine reduction rate at 25 °C, with specific activity expressed as enzyme units per milligram protein (U·mgprot-1). CAT activity was characterized as the enzyme amount catalyzing the decomposition of 1.0 μmol H_2_O_2_ per minute [39].

### 2.5. Identification of Genes Involved in Apoptosis and Immunity in Intestinal Samples

Intestinal samples were collected from four shrimp in each treatment group. Total RNA extraction from these tissues was performed using TRIzol, followed by reverse transcription into cDNA with the PrimeScript™ RT reagent kit, all according to the manufacturer’s instructions. The resulting cDNA was stored at −20 °C for subsequent use.

To assess the expression levels of immune-related genes, quantitative real-time PCR (qRT-PCR) was carried out on a CFX96™ Real-Time PCR Detection System using SYBR^®^ Premix Ex Taq™. The genes involved in apoptosis and immunity included those encoding caspase-3, Bcl-2, p53, TNF, TLRs, MyD88, hemocyanin, and ALF. The qRT-PCR conditions were as follows: an initial denaturation and polymerase activation step at 95 °C for 5 min, followed by 40 cycles of denaturation at 95 °C for 15 s, annealing, and extension at 60 °C for 45 s. A final melting curve analysis from 60 °C to 95 °C was conducted over 20 min to verify primer specificity and detect primer–dimers. Specific primers for each target gene were designed based on their coding sequences (Table 2) and synthesized by Shanghai Sangon Biotechnology Co., Ltd. (Shanghai, China). *β-actin* was used as the internal control gene. Relative gene expression levels were calculated using the 2^−ΔΔCt^ method [40].

### 2.6. Gut Microbiota Analysis

After exposure to alkalinity stress, the 0 mg/kg (CL) and 82.7 mg/kg (TL) MT treatment groups were selected for the determination of intestinal flora. Total DNA extracted from the gut samples was sequenced on the Illumina platform, with raw sequencing data saved in FASTQ format. For sequence processing, the DADA2 method was used within QIIME2 (version 2019.4, developed by the Caporaso Lab, Northern Arizona University, Flagstaff, AZ, USA) to perform depriming, quality filtering, denoising, splicing, and chimera removal. The experimental workflow had several key steps: First, the total microbiome DNA was extracted from the samples. This was followed by PCR amplification of the target gene fragments using Pfu high-fidelity DNA polymerase, ensuring consistent amplification conditions across all samples within each batch by strictly controlling the number of cycles. Next, PCR products were purified and recovered using magnetic beads and quantified via fluorescence. Sequencing libraries were prepared, leading to high-throughput sequencing. DNA quantification was carried out using a NanoDrop spectrophotometer (Thermo Fisher Scientific Inc., Waltham, MA, USA), and the quality of the extracted DNA was assessed through 1.2% agarose gel electrophoresis. The amplified PCR products were quantified using the Quant-iT PicoGreen dsDNA Assay Kit on a BioTek FLx800 Microplate reader (Agilent Technologies Inc., Santa Clara, CA, USA). Sequencing libraries were prepared using the TruSeq Nano DNA LT Library Prep Kit from Illumina.

After completing the denoising process for all libraries, amplicon sequence variant (ASV) feature sequences and tables were merged, and singletons (ASVs present only once in the entire data set) were removed according to default settings. Operational taxonomic units (OTUs) were clustered based on ASVs with a similarity threshold >97% and analyzed at a 70% confidence level. For α-diversity analysis, species richness was evaluated using Chao1 and Observed Species indices; diversity was measured using the Shannon and Simpson indices; evolution-based diversity was characterized by Faith’s Phylogenetic Diversity (PD) index; evenness was assessed using Pielou’s Evenness index; homogeneity was evaluated using the Goodness index; and coverage was determined by Good’s Coverage index. The Kruskal–Wallis H-test was used to test for between-group differences, followed by Dunn’s test for post hoc comparisons. Beta diversity analysis was interpreted using principal coordinates analysis (PCoA). Hierarchical clustering of data sets was performed using weighted UniFrac distances relative to species abundance. Community composition analyses were visualized through Venn diagrams generated using the VennDiagram package in R script (version 3.2.0; R Foundation for Statistical Computing, Vienna, Austria).

### 2.7. Statistical Analyses

All data are presented as means ± SEM. Data analysis was performed using SPSS 22.0 software (IBM Corporation, Armonk, NY, USA). One-way ANOVA followed by Duncan’s post hoc test was used for each variation in different environmental treatments, with significance set at *p* < 0.05. An independent samples *t*-test was used to detect differences between control and stress under the same MT dietary treatments, with significance set at * *p* < 0.05, ** *p* < 0.01, and *** *p* < 0.001. After correlation analysis and Mantel tests between the biochemical parameter matrix and the MT dietary factor matrix, the R package linkET was used to display associations between MT dietary and biochemical parameters, as well as correlations among the biochemical parameters, through network plots and correlation heat maps.

## 3. Results

### 3.1. Survival Rate and Morphology of Intestinal

After 96 h of acute alkalinity stress, there were significant differences between the groups fed different concentrations of melatonin (*p* < 0.05, Table 3). The survival rate of the 82.7 mg/kg group was the highest, with the survival rate of the 41.2 mg/kg and 165.1 mg/kg groups also being significantly higher than that of the control group (*p* < 0.05). However, there was no significant difference in terminal length and weight among the groups.

**Figure 1:**
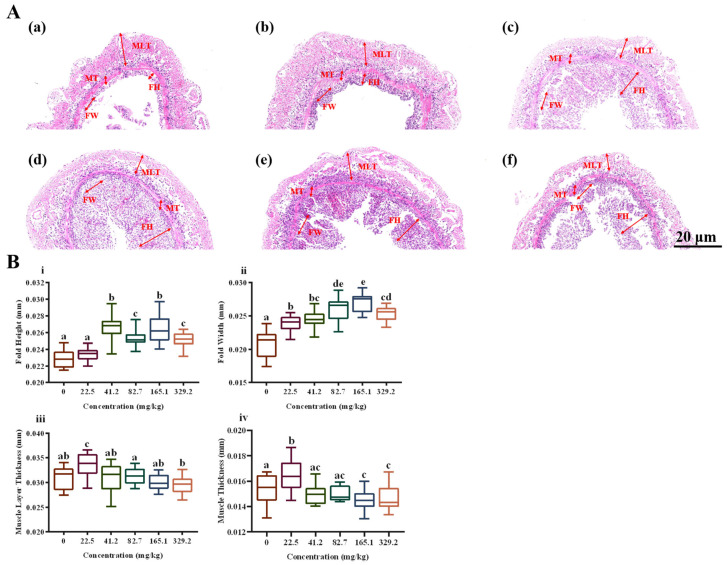
Effect of dietary melatonin levels on intestinal morphology of *L. vannamei* (paraffin-embedded tissue sections). (**A**) intestinal tissue morphology; (**B**) morphological parameters of intestinal tissue. Scale bars = 20 μm. FH, fold height; FW, fold width; MLT, muscle layer thickness; MT, muscle thickness. Different letters above the bars indicate significant differences (*p* < 0.05).

Histological analysis (Figure 1) showed that, under high alkalinity stress, the intestinal tissue morphology of *L. vannamei* varied significantly with the concentration of MT (*p* < 0.05). FH and FW were significantly higher in the high-concentration MT groups (41.2, 82.7, 165.1, and 329.2 mg/kg) compared with the control group (*p* < 0.05). MLT was maximally developed in the shrimp fed 22.5 mg/kg MT, which was significantly greater than that of the control group (*p* < 0.05), whereas no significant changes were observed in other treatment groups (*p* > 0.05). Compared with the control group, muscle thickness significantly decreased in the high-concentration MT groups (165.1 and 329.2 mg/kg) (*p* < 0.05) and significantly increased in the 22.5 mg/kg MT group (*p* < 0.05). Villus length in the intestine was notably reduced in the 0 mg/kg MT group compared with the 82.7 mg/kg MT group. In the 329.2 mg/kg MT group, the intestinal epithelium was almost completely detached from the basal lamina.

**Figure 2:**
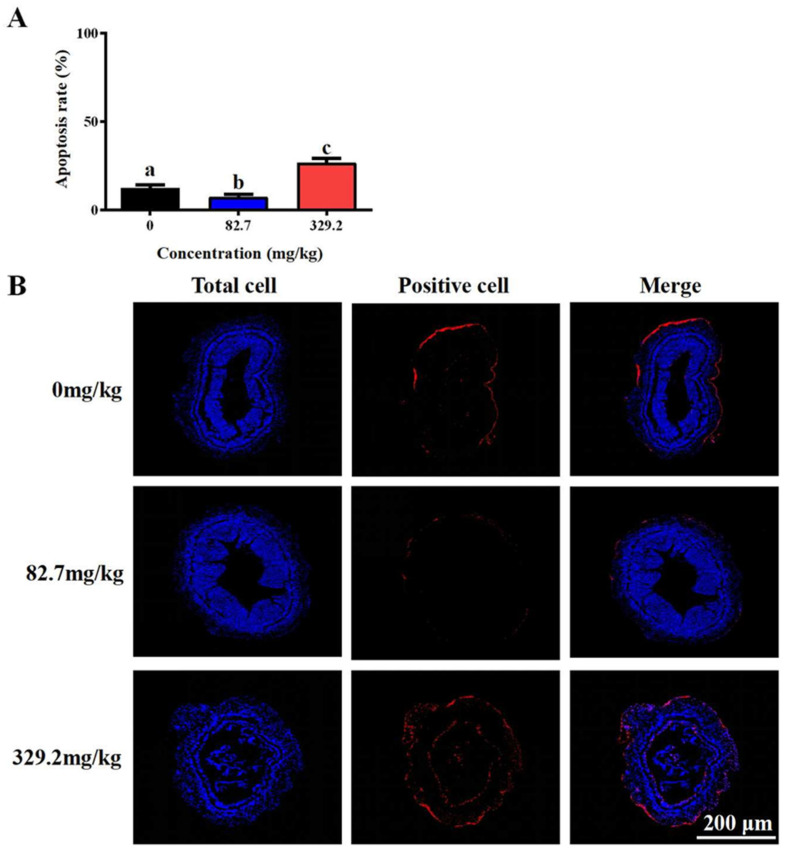
Effect of dietary melatonin levels on intestinal apoptosis of *L. vannamei*. (**A**) Apoptosis of intestinal tissues in the *L. vannamei* treatment groups (MT at 0, 82.7, and 329.2 mg/kg). Blue fluorescence indicates nuclei, and red fluorescence indicates apoptotic cells. (**B**) Apoptosis rates among groups. Different letters above the bars indicate significant differences (*p* < 0.05).

The proportion of apoptotic cells was significantly higher in the group fed 329.2 mg/kg MT compared with the control group (*p* < 0.05) (Figure 2A). Conversely, the group fed 82.7 mg/kg MT exhibited a significantly lower proportion of apoptotic cells relative to the control group (*p* < 0.05). Compared with the control group (0 mg/kg), the intestinal lumen structure was deformed in the 329.2 mg/kg MT group, whereas it remained well preserved in the 82.7 mg/kg MT group (Figure 2B).

### 3.2. Oxidative Stress-Related Indicators

**Figure 3:**
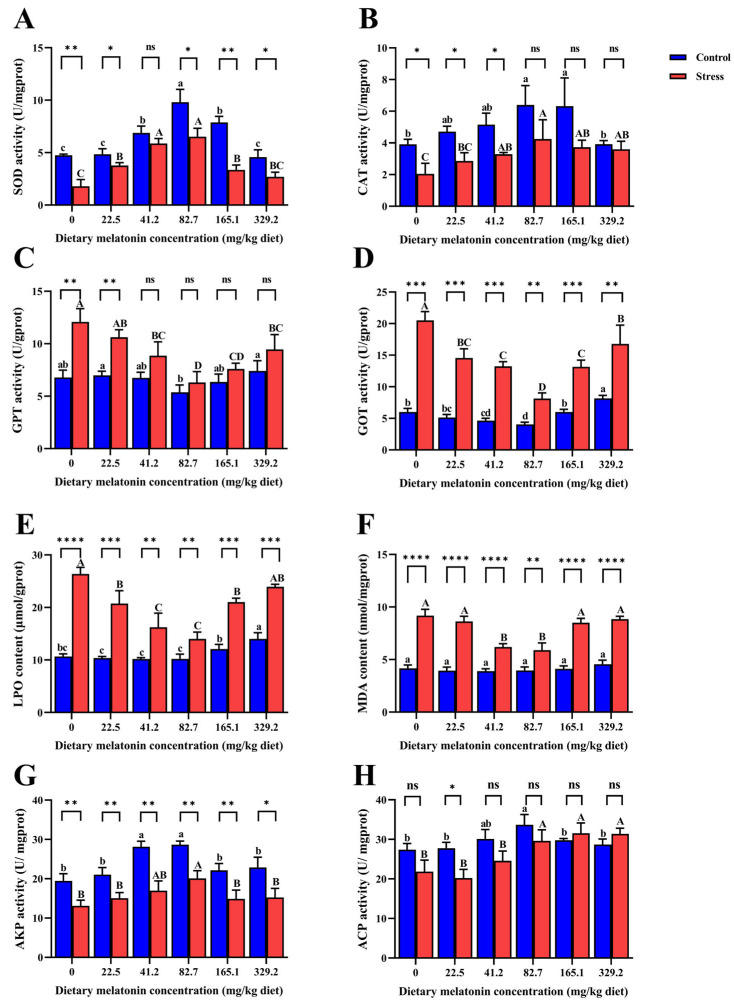
Effect of dietary melatonin levels on oxidative stress-related indicators in *L. vannamei*. (**A**) SOD (Superoxide dismutase) activity; (**B**) CAT (Catalase) activity; (**C**) GPT (Glutamate pyruvate transaminase) activity; (**D**) GOT (Glutamate oxaloacetate transaminase) activity; (**E**) LPO (Lipid peroxide) level; (**F**) MDA (Malondialdehyde) level; (**G**) AKP (Alkaline phosphatase) activity; (**H**) ACP (Acid phosphatase) activity. *p*-value (^ns^ *p* > 0.05, * p < 0.05, ** p < 0.01, *** p < 0.001, **** p < 0.0001). Different lowercase letters represent the significant differences among different concentrations of MT in the control group (*p* < 0.05), and different uppercase letters represent the significant differences among different concentrations of MT in the stress group (*p* < 0.05).

The results of oxidative stress-related indexes (Figure 3) revealed that, with the increase in MT concentration, SOD and CAT activities in the control and alkalinity stress groups first increased and then decreased. The enzyme activities in the 82.7 mg/kg and 165.1 mg/kg MT groups were significantly higher than in the 0 mg/kg MT group (*p* < 0.05). There was no significant difference in CAT activity between the control group and the alkalinity stress group in the 82.7 mg/kg, 165.1 mg/kg, and 329.2 mg/kg MT groups. Under alkalinity stress, the activities of GPT and GOT and the LPO and MDA content first increased and then decreased at higher MT concentrations. The lowest values of these four indexes occurred in the 82.7 mg/kg MT group and were significantly lower than those in the control group (*p* < 0.05). In the environment without alkalinity stress, there was no significant difference in MDA content among MT groups (*p* > 0.05). In addition, under alkalinity stress, the activities of AKP and ACP peaked in the 82.7 mg/kg MT group and were significantly higher than in the control group (*p* < 0.05); however, alkalinity stress did not significantly reduce the ACP activity in this treatment group (*p* > 0.05).

### 3.3. Gene Expressions

**Figure 4:**
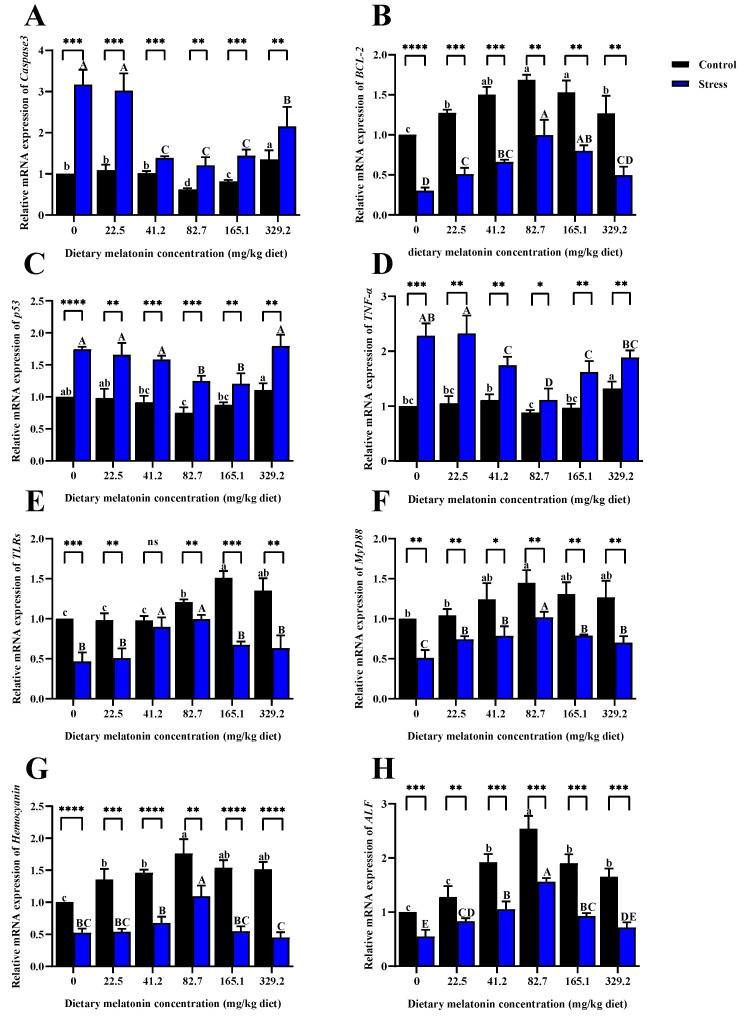
Effect of dietary melatonin levels on the expression levels of genes related to apoptosis and immune regulation in *L. vannamei*. (**A**) caspase-3 (cysteine-aspartic acid protease 3); (**B**) BCL-2 (B-cell lymphoma-2); (**C**) p53; (**D**) TNF-α (Tumor necrosis factor-alpha); (**E**) TLRs (Toll-like receptors); (**F**) MyD88 (Myeloid differentiation primary response 88); (**G**) Hemocyanin; (**H**) ALF (Anti-lipopolysaccharide factor). *p*-value (^ns^ *p* > 0.05, * p < 0.05, ** p < 0.01, *** p < 0.001, **** p < 0.0001). Different lowercase letters represent the significant differences among different concentrations of MT in the control group (*p* < 0.05), and different uppercase letters represent the significant differences among different concentrations of MT in the stress group (*p* < 0.05).

Analysis of the expression of apoptosis-related genes (Figure 4A–D) showed that, compared with the control alkalinity group, high alkalinity stress significantly upregulated the expression levels of *caspase-3*, *p53*, and *TNFa* (*p* < 0.05). However, the expression levels of these three genes in the 82.7 mg/kg and 165.1 mg/kg MT groups were significantly lower than in the 0 mg/kg MT group (*p* < 0.05). Alkalinity stress significantly decreased the expression level of *BCL2* in all groups (*p* < 0.05), with the highest value occurring in the 82.7 mg/kg MT group. Analysis of the expression of immune-related genes (Figure 4E–H) showed that, compared with the control alkalinity, high alkalinity stress significantly downregulated the expression levels of *TLRs*, *MyD88*, *hemocyanin*, and *ALF* (*p* < 0.05). However, the expression levels of these four genes in the 82.7 mg/kg MT group were significantly higher than in the 0 mg/kg MT group (*p* < 0.05).

### 3.4. Correlation Analysis

**Figure 5:**
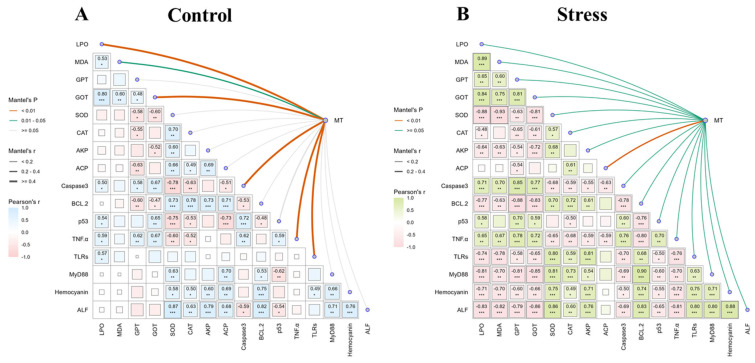
Ggcor correlation combination plots and regulatory network in *L. vannamei* at 24 h post alkalinity exposure. Indicator correlation in the (**A**) control group and (**B**) alkalinity stress group. Rows and columns correspond to the genes, and each cell contains the corresponding correlation and *p*-value (* p < 0.05, ** p < 0.01, *** p < 0.001). Pearson’s R-values are color-coded according to the color legend. The curve width corresponds to the mantel’s r statistic for the correlations between alkalinity exposure and indicators. The curve color corresponds to the mantel’s *p* statistic for the correlations between the alkalinity exposure and indicators.

A ggcor correlation chart was used to elucidate the potential regulatory mechanisms of MT (Figure 5). The control alkalinity results showed that different concentrations of melatonin significantly affected the content of LPO, activity of GOT, and gene expression levels of *caspase-3*, *TNFa*, and *TLR*. Moreover, SOD activity was positively correlated with the activity of antioxidant immuno-enzymes (CAT, AKP, and ACP) and negatively correlated with the gene expression levels of apoptosis factors (caspase-3, p53, and TNF-α). The results from the high alkalinity groups showed that different concentrations of melatonin were related only to ACP activity. In addition, antioxidant immune-related enzymes (SOD, CAT, AKP, and ACP) were significantly negatively correlated with oxidative stress indices (LPO, MDA, GPT, and GOT) and apoptosis factors (caspase-3 and TNF-α). However, there was a significant positive correlation with immune-related factors (TLRs, MyD88, hemocyanin, and ALF).

### 3.5. Sequencing of 16S rRNA and Annotation and Evaluation of Species

**Figure 6:**
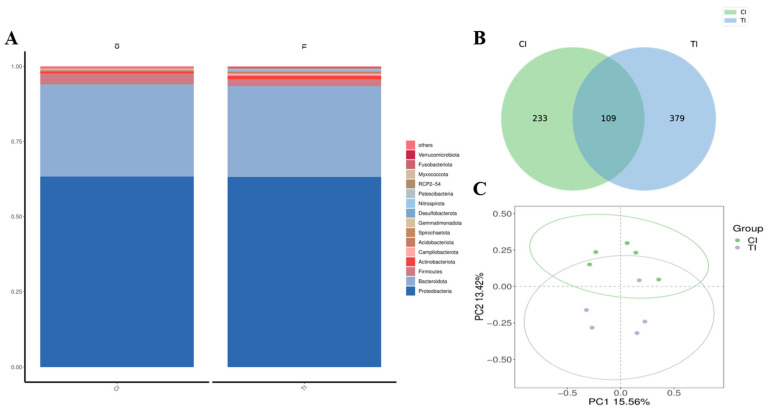
Impact of alkalinity stress on the gut microbiota of *L. vannamei* fed dietary MT [0 (Cl) and 82.7 (Tl) mg/kg] for 2 months. (**A**) Histogram of the relative distribution of the top 15 phyla (based on relative abundance) in each group. (**B**) Venn diagram of each group. (**C**) Principal coordinates analysis results.

After 2 months of dietary supplementation with MT, the intestinal flora of *L. vannamei* was analyzed based on phylum, class, order, family, and genus following exposure to high-alkalinity stress. Across all ten samples from both groups (0 and 82.7 mg/kg), most phylotypes belonged to four core phylum: *Proteobacteria* in group Cl (0.633) and group Tl (0.632); *Bacteroidota* in group Cl (0.31) and group Tl (0.3); *Firmicutes* in group Cl (0.036) and group Tl (0.023); and *Actinobacteriota* in group Cl (0.006) and group Tl (0.01) (Figure 6A). In *L. vannamei*, after high alkalinity stress, the distribution patterns of the three predominant phyla within each group were comparable across the MT concentration groups, although distinct differences were observed in both abundance and variation trends: there were 233 different species in group Cl and 379 different species in group Tl (Figure 6B). PCoA plots showed that, after high alkalinity stress, the groups fed MT at concentrations of 0 and 82.7 mg/kg exhibited significant clustering, suggesting a favorable grouping effect (Figure 6C).

### 3.6. Analysis of Species and Genera

**Figure 7:**
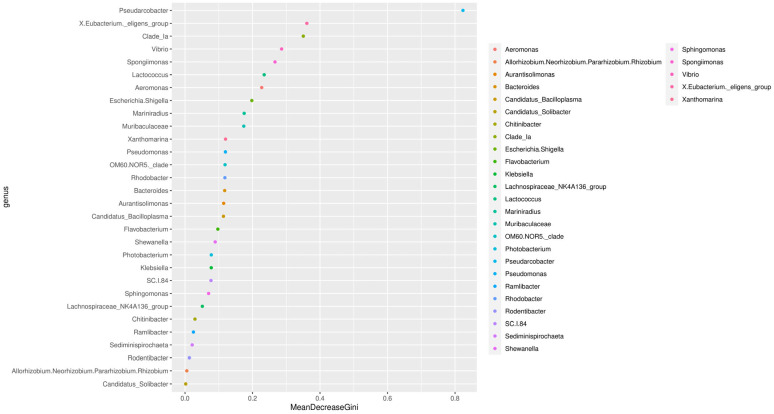
Random forest analysis based on genus level of gut microbiota of *L. vannamei* fed dietary MT [0 (Cl) and 82.7 (Tl) mg/kg] for 2 months and then exposed to high alkalinity stress.

**Figure 8:**
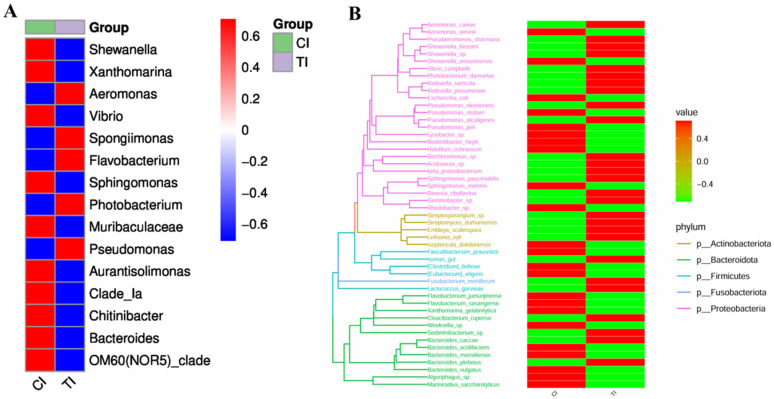
Impact of alkalinity stress on the gut microbiota in *L. vannamei* fed dietary MT [0 (Cl) and 82.7 (Tl) mg/kg] for 2 months. (**A**) Heat map of differences between the two groups at the genus level. Sample information is on the horizontal axis, and species labeling information is on the vertical axis. Higher relative abundance is in red, and lower relative abundance is in blue. (**B**) Phylogenetic tree between populations and heat map of species abundance in both groups. The evolutionary tree is shown on the left. Different colors of the branches represent different phyla. The tip of each branch is an operational taxonomic unit (OTU).

The results of random forest algorithms showed that *Pseudarcobacter*, *Eubacterium*, *Vibrio*, and *Spongiimonas* were the key components of the differences caused by the addition of MT after alkalinity stress (Figure 7). After feeding with 82.7 mg/kg of MT, the relative abundance of *Aeromonas*, *Spongiimonas*, *Flavobacterium*, *Photobacterium*, and *Pseudomonas* was high in the gut following exposure to high-alkalinity stress (Figure 8A). The abundance of *Shewanella bicestrii*, *Dechloromonas* sp., *Acidovorax* sp., *Sphingomonas paucimobilis*, *Gemmobacter* sp., *Streptomyces durhamensis*, and *Cloacibacterium rupense* in the gut of *L. vannamei* fed 82.7 mg/kg MT also increased (Figure 8B). Compared with the control group, the relative abundance of *Escherichia coli*, *Pseudomonas stutzeri*, *Rodentibacter heylii*, and *Weeksella* sp. in the intestinal tract decreased with MT supplementation.

## 4. Discussion

The increasing impact of alkaline stress on aquaculture has drawn research attention to dietary additives. Some studies have found that adding dietary melatonin can improve the ability of Chinese mitten crab to resist ammonia nitrogen stress [41] and that melatonin supplementation can promote the growth and lipid utilization of *P. vannamei* [42]. The results of the current study revealed that the survival rate of *L. vannamei* fed a diet supplemented with melatonin was significantly improved after 96 h of acute alkalinity stress, indicating that melatonin improves the stress resistance of shrimp and, thus, enhances their survival [43].

Pacific white shrimp show increased reactive oxygen species (ROS) levels in gills and hepatopancreas under alkalinity stress [44]. Excessive ROS can cause damage to the body, such as intensifying lipid peroxidation, resulting in tissue damage [45]. Organisms have an antioxidant defense system, which includes SOD and CAT, to remove excess oxygen free radicals [46]. SOD and CAT are essential antioxidant enzymes found in almost all living organisms and can counteract the effects of oxidative stress by eliminating H_2_O_2_ [47,48]. In this study, the activity of antioxidant enzymes showed a similar trend before and after high alkali stress, and their activity was high under optimal dietary melatonin supplementation. Previous research showed that high alkaline stress can lead to acute oxidative stress in shrimp [49]. And the research by Zhang et al. [50] indicates that the *Eriocheir sinensis* initiates anti-inflammatory effects and excretory regulation to resist stress under high alkalinity stress. However, this study showed that the addition of MT can help reduce the damage to the organism. LPO and MDA are used to measure the degree of oxidative damage to the body [51]. The LPO and MDA activity levels in the stress group were significantly higher than in the control group, indicating that the degree of lipid peroxidation damage after high alkali stress in shrimp was alleviated by the addition of melatonin. Some authors reported that exogenous melatonin supplementation can reduce oxidative damage to the body [52]. As an oxygen free radical scavenger, melatonin itself exerts an effect on reducing ROS content, which also indicates that it can help alleviate high alkali stress [53].

Some metabolic processes in aquatic organisms are catalyzed by different kinds of phosphatase [54]. For example, ACP and AKP are involved in the transfer and metabolism of phosphate groups and might also have a crucial role in immune defense; thus, they are often chosen as indicators that reflect the immune status of aquatic animals [55]. In this study, melatonin significantly increased the activity of AKP and ACP after acute high alkali exposure, indicating that melatonin can promote immune activity under high alkali stress, which is consistent with other reported results [42]. GOT and GPT are used to assess the degree of liver damage and are usually released into the hemolymph when the liver is damaged [56,57]. This study found that melatonin significantly reduced the content of GOT and GPT under high alkali stress, indicating that melatonin has a certain protective role in hepatopancreas and improved stress resistance.

Apoptosis is the programmed death of cells and is usually used to remove senescent, damaged cells from living organisms [58]. The process is regulated by a variety of genes. For example, the functions of p53 include cell cycle regulation [59]. If DNA damage is severe or irreparable, p53 also induces apoptosis [60]. The effector caspases, caspase-3, -6, and -7, directly cleave key cellular protein substrates, leading to apoptosis [61]. Notably, melatonin exhibits a concentration-dependent dual role in apoptosis regulation. While physiological concentrations of melatonin typically exert antioxidant and anti-apoptotic effects, supraphysiological doses may paradoxically promote apoptosis through pro-oxidant activity, a biphasic behavior that could be attributed to the generation of reactive oxygen species when melatonin exceeds optimal concentrations [62]. In the current study, compared with before alkaline stress, the expression of caspase and p53 increased, indicating that high alkali stress induced an apoptosis response; this was the lowest in the optimal melatonin group. These results indicate that the appropriate addition of melatonin can reduce the level of apoptosis and that it has a protective effect [58].

The Toll pathway has a key role in regulating invertebrate innate immune responses, and MyD88 is a key molecule in this signaling pathway [63]. Toll-like receptors (TLRs) are pattern recognition molecules that are the first line of defense against pathogen invasion [64]. We specifically evaluated three categories of immune markers: TNFα (a pro-inflammatory cytokine), a member of tumor necrosis factor, has a role in the early immune response of shrimp to inflammation [65]; the antimicrobial peptide represented by anti-lipopolysaccharide factor (ALF) is an antimicrobial peptide (AMP) with a vital role in antimicrobial defense [66]; and pattern recognition receptors, including the aforementioned TLRs. These selected markers are known to be stress-responsive in crustacean immunity systems. In this study, all three categories of immune markers showed downregulated expression after high alkali stress compared with pre-stress levels, indicating that high alkali conditions suppressed both pathogen recognition (via TLRs), antimicrobial defense (via ALF), and inflammatory regulation (via TNFα). Notably, their expression was strongest in the optimal melatonin group, suggesting that melatonin might activate multiple defense mechanisms including cytokine production, antimicrobial peptide synthesis, and pattern recognition. This is particularly relevant to the stress response as melatonin has been shown to enhance the production of various cytokines (e.g., TNFα) in macrophages, natural killer (NK) cells, and T lymphocytes, while regulating intracellular glutathione levels [67,68,69], mechanisms that collectively improve stress tolerance through immunomodulation.

The intestine is an important digestive and immune organ in aquatic organisms, serving as a crucial defense barrier against the invasion of external pathogenic substances [25]. The steady-state balance of the gut microbiota has an important role in maintaining the health and survival of aquatic organisms. Exogenous supplementation of specific nutrients can affect the homeostasis of the inner environment of the intestinal flora [70]. Proteobacteria, which are responsible for breaking down polysaccharides, proteins, and other organic matter, are the dominant group in the gut microbiota of many animals [71]. Our analysis showed that higher MT pretreatment significantly increased the abundance of *Proteobacteria* after high alkaline stress relative to the group without MT addition, suggesting that the growth conditions of *Proteobacteria* affect the gut health of shrimps. Furthermore, Vibrio species (known for chitin degradation capacity [72]) and Pseudomonas spp. (capable of producing short-chain fatty acids [73]) were markedly enriched in MT-treated groups. These taxa have been reported to enhance intestinal barrier function and pathogen resistance in crustaceans [74], suggesting that MT-induced Proteobacteria proliferation may improve gut health through both nutritional and immunological pathways. Many members of the *Firmicutes* and *Bacteroidetes* are involved in the degradation of polysaccharides in organisms [75]. Notably, the firmicute/bacteroidetoid ratio is associated with obesity and efficient energy absorption from food, as well as with improved shrimp growth performance [76]. Therefore, melatonin might improve the anti-stress response by improving the intestinal immune state. Many studies have shown that melatonin can improve biological immunity. For example, melatonin supplementation can activate the expression of immune-related genes, including those encoding anti-inflammatory factors [16]. Melatonin is also involved in the regulation of T lymphocytes and the activation of B cells to enhance immune function [77]. Therefore, improving the immune capacity of aquatic animals with dietary melatonin can be an efficient and practical method for the aquaculture industry.

The environmental profile of melatonin shows promise through rapid photodegradation (≤48 h half-life under UV), yet its dose-dependent modulation of Proteobacteria dominance necessitates long-term monitoring of antibiotic resistance gene transfer in aquaculture ecosystems. Three key limitations constrain the current findings: 1. Acute 96 h exposure models inadequately replicate chronic alkalinity fluctuations in coastal rearing systems. 2. Unresolved melatonin metabolite pathways in hemolymph prevent full biodegradation assessment. 3. Fixed feeding regimes (5% body weight/day) neglect natural appetite variations during stress cycles. These constraints underscore the imperative for longitudinal studies to verify melatonin’s ecological safety, particularly regarding microbial community resilience and photodegradation byproduct persistence across seasonal conditions.

## 5. Conclusions

This study investigated the effects of melatonin supplementation on the intestinal physiology, histology, and transcriptome of *L. vannamei* under acute alkaline stress. The results showed that dietary supplementation of melatonin could effectively improve the tolerance of *L. vannamei* to high alkalinity conditions. Melatonin was able to alleviate damage to the intestinal tissue structure induced by alkalinity stress and reduce cell apoptosis as well as alleviate intestinal oxidative stress and enhance immune performance. In addition, the number of beneficial intestinal bacteria was significantly increased. This study details a preliminary explanation of the mechanism of action of melatonin in alleviating high alkali stress, providing a scientific basis for precision nutrition regulation to improve saline–alkali shrimp aquaculture.

## Figures and Tables

**Table 1:** Feed formulation and proximate composition of the basal feed (dry matter basis).

Ingredients	g kg^−1^ Diet
Casein	450
Wheat flour	260
Gelatin	80
Fish oil	40
Soybean oil	20
Soyabean lecithin	10
Vitamin premix ^a^	20
Mineral premix ^b^	20
Choline chloride	5
Cholesterol	5
Carboxy methyl cellulose	20
Alpha-cellulose	70
Total	1000
Proximate composition	
Crude protein	37.27
Crude lipid	10.12
Crude ash	3.45
Moisture	10.28
Melatonin basic content ^c^ (mg/kg)	ND

^a^ Vitamin premix (mg kg^−1^ premix): microorganism A, 1000 mg; Vitamin D3, 300 mg; Vitamin E, 2000 mg; Vitamin K3, 600 mg; Vitamin C, 6000 mg; Vitamin B1, 1000 mg; Microbial B2, 1000 mg; Vitamin B6, 1200 mg; Vitamin B12, 200 mg; Calcium pantothenate (vitamin B5), 3000 mg; Biotin, 500 mg; Folic acid, 300 mg; Niacin (microbial B3), 4000 mg; Myo-inositol, 6000 mg. ^b^ Mineral premix (mg kg^−1^ premix): zinc sulfate monohydrate, 41.17; Calcium iodate, 0.234; Copper sulfate pentahydrate, 1.25; Manganese sulfate monohydrate, 3.25; Magnesium sulfate monohydrate, 79.72; Cobalt chloride, 0.02; Ferrous sulfate monohydrate, 22.358; Sodium selenite, 0.05; Calcium hydrogen phosphate, 332.884; Zeolite powder, 519.064. ^c^ ND: undetected. The basic melatonin content was below the detectable range.

**Table 2:** Nucleotide sequences and sources of primers for qRT-PCR analysis.

Primer Name	Primer Sequence (5′–3′)	NCBI Database, Gene Accession Number
*Caspase-3 F*	AGTTAGTACAAACAGATTGGAGCG	KC660103.1
*Caspase-3 R*	TTGTGGACAGACAGTATGAGGC	
*BCL-2 F*	CCTTGCTTGACACAGTCGGA	MH559339.1
*BCL-2 R*	CAGACAAGGTCGTGAGGTGG	
*p53 F*	CCAAGCAGCAATGTGTCAG	KX179650.1
*P53 R*	CTTGTTGCGATCTTTGTTGC	
*TNF-α F*	CTCAGCCATCTCCTTCTTG	JN180639.1
*TNF-α R*	TGTTCTCCTCGTTCTTCAC	
TLRs *F*	CCAGCTTAGAAGACCGGCAA	KT372179.1
TLRs *R*	GTTGTCCGAGCAGAAGTCCA	
*MyD88 F*	GCTGTTCCACCGCCATTT	JX073568.1
*MyD88 R*	GCATCATAGTGCTGTAGTCCAAGA	
*Hemocyanin F*	GTCTTAGTGGTTCTTGGGCTTGTC	KJ151291.1
*Hemocyanin R*	GGTCTCCGTCCTGAATGTCTCC	
*ALF F*	AGAGGATCGTTGGGTTGTGG	KJ000049.1
*ALF R*	AATTCTAGCGTCGTCCTCCG	
*β-actin F*	CAGGTCGTGACTTGACCGAT	KY780290
*β-actin R*	CGTCAGGGAGCTCGTAAGAC	

**Table 3:** Effect of dietary melatonin levels on the growth performance of *L. vannamei*. SR: survival rate.

Dietary Melatonin Level (mg/kg)	Parameters				
	SR (%)	Initial Weight (g)	Initial Length (cm)	Terminal Weight (g)	Terminal Length (cm)
0	41.67 ± 6.24 ^c^	3.86 ± 0.63	6.08 ± 0.32	3.87 ± 0.46	6.12 ± 0.26
22.5	48.33 ± 6.24 ^bc^	3.86 ± 0.63	6.08 ± 0.32	3.95 ± 0.49	6.15 ± 0.21
41.2	60 ± 4.08 ^ab^	3.86 ± 0.63	6.08 ± 0.32	3.96 ± 0.88	6.11 ± 0.37
82.7	68.33 ± 4.71 ^a^	3.86 ± 0.63	6.08 ± 0.32	3.93 ± 0.58	6.19 ± 0.28
165.1	58.33 ± 6.24 ^ab^	3.86 ± 0.63	6.08 ± 0.32	3.96 ± 0.48	6.12 ± 0.3
329.2	51.67 ± 2.36 ^bc^	3.86 ± 0.63	6.08 ± 0.32	3.9 ± 0.3	6.11 ± 0.26

Note: different letters above the bars indicate significant differences (*p* < 0.05).

## Data Availability

The authors declare that all data supporting the conclusions of this study are available within the article.

## References

[1] Mannino G., Pernici C., Serio G., Gentile C., Bertea C.M. (2021). Melatonin and Phytomelatonin: Chemistry, Biosynthesis, Metabolism, Distribution and Bioactivity in Plants and Animals—An Overview. Int. J. Mol. Sci..

[2] Rao K.S.J., Hegde M.L., Anitha S., Musicco M., Zucca F.A., Turro N.J., Zecca L. (2006). Amyloid beta and neuromelanin–toxic or protective molecules? The cellular context makes the difference. Prog. Neurobiol..

[3] Ángeles Esteban M., Cuesta A., Chaves-Pozo E., Meseguer J. (2013). Influence of melatonin on the immune system of fish: A review. Int. J. Mol. Sci..

[4] Yasmin F., Sutradhar S., Das P., Mukherjee S. (2021). Gut melatonin: A potent candidate in the diversified journey of melatonin research. Gen. Comp. Endocrinol..

[5] Tilden A.R., Brauch R., Ball R., Janze A.M., Ghaffari A.H., Sweeney C.T., Yurek J.C., Cooper R.L. (2003). Modulatory effects of melatonin on behavior, hemolymph metabolites, and neurotransmitter release in crayfish. Brain Res..

[6] Pape C., Teschke M., Meyer B. (2008). Melatonin and its possible role in mediating seasonal metabolic changes of Antarctic krill, *Euphausia superba*. Comp. Biochem. Physiol. Part A Mol. Integr. Physiol..

[7] Sainath S.B., Reddy P.S. (2010). Evidence for the involvement of selected biogenic amines (serotonin and melatonin) in the regulation of molting of the edible crab, Oziotelphusa senex senex Fabricius. Aquaculture.

[8] Maciel F.E., Geihs M.A., Cruz B.P., Vargas M.A., Allodi S., Marins L.F., Nery L.E.M. (2014). Melatonin as a signaling molecule for metabolism regulation in response to hypoxia in the crab *Neohelice granulata*. Int. J. Mol. Sci..

[9] Maciel F.E., Geihs M.A., Vargas M.A., Cruz B.P., Ramos B.P., Vakkuri O., Meyer-Rochow V.B., Maia Nery L.E., Allodi S. (2008). Daily variation of melatonin content in the optic lobes of the crab *Neohelice granulata*. Comp. Biochem. Physiol. Part A Mol. Integr. Physiol..

[10] Chen S., Migaud H., Shi C., Song C., Wang C., Ye Y., Ren Z., Wang H., Mu C. (2021). Light intensity impacts on growth, molting and oxidative stress of juvenile mud crab *Scylla paramamosain*. Aquaculture.

[11] Reiter R.J., Tan D.X., Osuna C., Gitto E. (2000). Actions of melatonin in the reduction of oxidative stress: A review. J. Biomed. Sci..

[12] Galano A., Tan D.X., Reiter R.J. (2011). Melatonin as a natural ally against oxidative stress: A physicochemical examination. J. Pineal Res..

[13] Hardeland R., Pandi-Perumal S.R., Cardinali D.P. (2006). Melatonin. Int. J. Biochem. Cell Biol..

[14] Yang Y., Xu W., Du X., Ye Y., Tian J., Li Y., Jiang Q., Zhao Y. (2023). Effects of dietary melatonin on growth performance, antioxidant capacity, and nonspecific immunity in crayfish, *Cherax destructor*. Fish Shellfish Immunol..

[15] Li Y., Ye Y., Li S., Feng J., Liu X., Che X., Jiang Q., Chen X. (2023). Transcriptomic analysis of the antioxidant responses and immunomodulatory effects of dietary melatonin in red swamp crayfish (*Procambarus clarkii*). Fish Shellfish Immunol..

[16] Yang X., Song X., Zhang C., Pang Y., Song Y., Cheng Y., Nie L., Zong X. (2020). Effects of dietary melatonin on hematological immunity, antioxidant defense and antibacterial ability in the Chinese mitten crab, *Eriocheir sinensis*. Aquaculture.

[17] Shang X., Geng L., Yang J., Zhang Y., Xu W. (2021). Transcriptome analysis reveals the mechanism of alkalinity exposure on spleen oxidative stress, inflammation and immune function of *Luciobarbus capito*. Ecotoxicol. Environ. Saf..

[18] Fan Z., Wu D., Li J., Li C., Zheng X., Wang L. (2022). Phosphorus Nutrition in Songpu Mirror Carp (*Cyprinus carpio* Songpu) During Chronic Carbonate Alkalinity Stress: Effects on Growth, Intestinal Immunity, Physical Barrier Function, and Intestinal Microflora. Front. Immunol..

[19] Song Z., Li K., Li K. (2024). Integrated characterizations of intestinal bacteria and transcriptomics revealed the acute stress response to carbonate alkalinity in white shrimp *Penaeus vannamei*. Fish Shellfish Immunol..

[20] Zhang R., Shi X., Guo J., Mao X., Fan B. (2024). Acute stress response in gill of Pacific white shrimp *Litopenaeus vannamei* to high alkalinity. Aquaculture.

[21] Zhang R., Shi X., Guo J., Mao X., Fan B. (2024). Acute stress response in hepatopancreas of Pacific white shrimp Litopenaeus vannamei to high alkalinity. Aquac. Rep..

[22] Zhang R., Zhao Z., Li M., Luo L., Wang S., Guo K., Xu W. (2023). Effects of saline-alkali stress on the tissue structure, antioxidation, immunocompetence and metabolomics of *Eriocheir sinensis*. Sci. Total Environ..

[23] Li W., Wang J., Li J., Liu P., Fei F., Liu B., Li J. (2024). The effect of astaxanthin on the alkalinity stress resistance of Exopalaemon carinicauda. Sci. Total Environ..

[24] Wei X., Wu H., Wang Z., Zhu J., Wang W., Wang J., Wang Y., Wang C. (2023). Rumen-protected lysine supplementation improved amino acid balance, nitrogen utilization and altered hindgut microbiota of dairy cows. Anim. Nutr..

[25] Medina-Félix D., Garibay-Valdez E., Vargas-Albores F., Martínez-Porchas M. (2023). Fish disease and intestinal microbiota: A close and indivisible relationship. Rev. Aquac..

[26] Yan Y., Li B., Gao Q., Wu M., Ma H., Bai J., Ma C., Xie X., Gong Y., Xu L. (2025). Intestine-Decipher Engineered Capsules Protect Against Sepsis-induced Intestinal Injury via Broad-spectrum Anti-inflammation and Parthanatos Inhibition. Adv. Sci..

[27] Guo K., Ruan G., Fan W., Wang Q., Fang L., Luo J., Liu Y. (2020). Immune response to acute heat stress in the intestine of the red swamp crayfish, *Procambarus clarkii*. Fish Shellfish Immunol..

[28] Zhou Z., Ding Z., Huiyuan L.V. (2007). Effects of Dietary Short-chain Fructooligosaccharides on Intestinal Microflora, Survival, and Growth Performance of Juvenile White Shrimp, *Litopenaeus vannamei*. J. World Aquac. Soc..

[29] Chen F., Wang Y., Wang K., Chen J., Jin K., Peng K., Chen X., Liu Z., Ouyang J., Wang Y. (2023). Effects of Litsea cubeba essential oil on growth performance, blood antioxidation, immune function, apparent digestibility of nutrients, and fecal microflora of pigs. Front. Pharmacol..

[30] Pan S., Hong F., Li L., Guo Y., Qiao X., Zhang J., Xu P., Zhai Y. (2021). Melatonin Attenuates Dextran Sodium Sulfate Induced Colitis in Obese Mice. Pharmaceuticals.

[31] Velarde E., Alonso-Gómez Á.L., Azpeleta C., Isorna E., De Pedro N., Delgado M.J. (2011). Melatonin effects on gut motility are independent of the relaxation mediated by the nitrergic system in the goldfish. Comp. Biochem. Physiol. Part A Mol. Integr. Physiol..

[32] Miao Z., Zhao P., Cao Q., Ding Y., Xu S. (2024). Protective effect of melatonin on imidacloprid-induced pyroptosis and ferroptosis by mediating peptidoglycan in the gut of the common carp (*Cyprinus carpio*). Pestic. Biochem. Physiol..

[33] Song Y., Song X., Wu M., Pang Y., Shi A., Shi X., Niu C., Cheng Y., Yang X. (2020). The protective effects of melatonin on survival, immune response, digestive enzymes activities and intestinal microbiota diversity in Chinese mitten crab (*Eriocheir sinensis*) exposed to glyphosate. Comp. Biochem. Physiol. Toxicol. Pharmacol..

[34] Ahmadi S., Taghizadieh M., Mehdizadehfar E., Hasani A., Khalili Fard J., Feizi H., Hamishehkar H., Ansarin M., Yekani M., Memar M.Y. (2024). Gut microbiota in neurological diseases: Melatonin plays an important regulatory role. Biomed. Pharmacother..

[35] Wyban J. (2019). Selective Breeding of *Penaeus vannamei:* Impact on World Aquaculture and Lessons for Future. J. Coast. Res..

[36] Yuan H., Xie M., Chen J., Hu N., Wang H., Tan B., Shi L., Zhang S. (2024). Combined intestinal microbiota and transcriptomic analysis to investigate the effect of different stocking densities on the ability of Pacific white shrimp (*Litopenaeus vannamei*) to utilize Chlorella sorokiniana. Anim. Nutr..

[37] Tendencia E.A., de la Peña L.D. (2001). Antibiotic resistance of bacteria from shrimp ponds. Aquaculture.

[38] Mardones O., Devia E., Labbé B.S., Oyarzún R., Vargas-Chacoff L., Muñoz J.L.P. (2018). Effect of l-tryptophan and melatonin supplementation on the serotonin gastrointestinal content and digestive enzymatic activity for Salmo salar and Oncorhynchus kisutch. Aquaculture.

[39] Liu X.-L., Xi Q.-Y., Yang L., Li H.-Y., Jiang Q.-Y., Shu G., Wang S.-B., Gao P., Zhu X.-T., Zhang Y.-L. (2011). The effect of dietary Panax ginseng polysaccharide extract on the immune responses in white shrimp, *Litopenaeus vannamei*. Fish Shellfish Immunol..

[40] Livak K.J., Schmittgen T.D. (2001). Analysis of Relative Gene Expression Data Using Real-Time Quantitative PCR and the 2^−ΔΔCT^ Method. Methods.

[41] Yang X., Shi A., Song Y., Niu C., Yu X., Shi X., Pang Y., Ma X., Cheng Y. (2021). The effects of ammonia-N stress on immune parameters, antioxidant capacity, digestive function, and intestinal microflora of Chinese mitten crab, *Eriocheir sinensis*, and the protective effect of dietary supplement of melatonin. Comp. Biochem. Physiol. Part C Toxicol. Pharmacol..

[42] Ye Y., Li S., Zhu B., Yang Y., Du X., Li Y., Zhao Y. (2024). Effects of dietary melatonin on growth performance, nutrient composition, and lipid metabolism of Pacific white shrimp (*Penaeus vannamei*). Aquaculture.

[43] Li Y., Yang Y., Li S., Ye Y., Du X., Liu X., Jiang Q., Che X. (2023). Effects of dietary melatonin on antioxidant and immune function of the Pacific white shrimp (*Litopenaeus vannamei*), as determined by transcriptomic analysis. Comp. Biochem. Physiol. Part D Genom. Proteom..

[44] Zhang R., Shi X., Liu Z., Sun J., Sun T., Lei M. (2023). Histological, Physiological and Transcriptomic Analysis Reveal the Acute Alkalinity Stress of the Gill and Hepatopancreas of *Litopenaeus vannamei*. Mar. Biotechnol..

[45] Wang B., Feng L., Chen G.F., Jiang W.D., Liu Y., Kuang S.Y., Jiang J., Tang L., Wu P., Tang W.N. (2016). Jian carp (*Cyprinus carpio* var. Jian) intestinal immune responses, antioxidant status and tight junction protein mRNA expression are modulated via Nrf2 and PKC in response to dietary arginine deficiency. Fish Shellfish Immunol..

[46] Wang B., Liu Y., Feng L., Jiang W.D., Kuang S.Y., Jiang J., Li S., Tang L., Zhou X.Q. (2015). Effects of dietary arginine supplementation on growth performance, flesh quality, muscle antioxidant capacity and antioxidant-related signalling molecule expression in young grass carp (*Ctenopharyngodon idella*). Food Chem..

[47] Campa-Córdova A.I., Hernández-Saavedra N.Y., De Philippis R., Ascencio F. (2002). Generation of superoxide anion and SOD activity in haemocytes and muscle of American white shrimp (*Litopenaeus vannamei*) as a response to β-glucan and sulphated polysaccharide. Fish Shellfish Immunol..

[48] Xu Z., Regenstein J.M., Xie D., Lu W., Ren X., Yuan J., Mao L. (2018). The oxidative stress and antioxidant responses of *Litopenaeus vannamei* to low temperature and air exposure. Fish Shellfish Immunol..

[49] Shi X., Zhang R., Liu Z., Sun J., Li L., Zhao G., Lu J. (2023). Combined analysis of mRNA and miRNA reveals the mechanism of pacific white shrimp (*Litopenaeus vannamei*) under acute alkalinity stress. PLoS ONE.

[50] Zhang R., Zhao Z., Li M., Luo L., Wang S., Guo K., Xu W. (2023). Metabolomics analysis reveals the response mechanism to carbonate alkalinity toxicity in the gills of *Eriocheir sinensis*. Comp. Biochem. Physiol. Part C Toxicol. Pharmacol..

[51] Duan Y., Wang Y., Zhang J., Liu Q., Ding X. (2018). Morphologic, digestive enzymes and immunological responses of intestine from *Litopenaeus vannamei* after lipopolysaccharide injection. J. Invertebr. Pathol..

[52] Geihs M.A., Vargas M.A., Maciel F.E., Caldas S.S., Cruz B.P., Primel E.G., Monserrat J.M., Nery L.E.M. (2010). Effect of melatonin in the antioxidant defense system in the locomotor muscles of the estuarine crab *Neohelice granulata* (Decapoda, Brachyura). Gen. Comp. Endocrinol..

[53] Zhang C., Zhang Q., Pang Y., Song X., Zhou N., Wang J., He L., Lv J., Song Y., Cheng Y. (2019). The protective effects of melatonin on oxidative damage and the immune system of the Chinese mitten crab (*Eriocheir sinensis*) exposed to deltamethrin. Sci. Total Environ..

[54] Dai X., Zhang L.-T., Zang W.-L., Deng P.-P., Zou W.-L., Ding F. (2012). Effect of Ca~(2+), Mg~(2+) and salinity on survival, growth and shrimp taste of *Litopenaeus vannamei*. J. Fish. China.

[55] Xue J., Xu Y., Jin L., Liu G., Sun Y., Li S., Zhang J. (2008). Effects of traditional Chinese medicine on immune responses in abalone, *Haliotis discus* hannai Ino. Fish Shellfish Immunol..

[56] Hossain M.S., Koshio S., Ishikawa M., Yokoyama S., Sony N.M. (2016). Dietary effects of adenosine monophosphate to enhance growth, digestibility, innate immune responses and stress resistance of juvenile red sea bream, *Pagrus major*. Fish Shellfish Immunol..

[57] Zhou J., Wang Y.H., Chu J., Luo L.Z. (2008). Improvement of innate immune responses and defense activity in mitten crab (*Eriocheir sinensis*) by oral administration of beta-glucan. Biotechnol. Lett..

[58] Liang Z., Liu R., Zhao D., Wang L., Sun M., Wang M., Song L. (2016). Ammonia exposure induces oxidative stress, endoplasmic reticulum stress and apoptosis in hepatopancreas of pacific white shrimp (*Litopenaeus vannamei*). Fish Shellfish Immunol..

[59] Prives C. (1998). Signaling to p53: Breaking the MDM2–p53 Circuit. Cell.

[60] Ko L.J., Prives C. (1996). p53: Puzzle and paradigm. Genes Dev..

[61] Houston A., O’Connell J. (2004). The Fas signalling pathway and its role in the pathogenesis of cancer. Curr. Opin. Pharmacol..

[62] Zhang H.-M., Zhang Y. (2014). Melatonin: A well-documented antioxidant with conditional pro-oxidant actions. J. Pineal Res..

[63] Deepika A., Sreedharan K., Paria A., Makesh M., Rajendran K.V. (2014). Toll-pathway in tiger shrimp (*Penaeus monodon*) responds to white spot syndrome virus infection: Evidence through molecular characterisation and expression profiles of MyD88, TRAF6 and TLR genes. Fish Shellfish Immunol..

[64] Dechamma M.M., Rajeish M., Maiti B., Mani M.K., Karunasagar I. (2015). Expression of Toll-like receptors (TLR), in lymphoid organ of black tiger shrimp (*Penaeus monodon*) in response to *Vibrio harveyi* infection. Aquac. Rep..

[65] Mekata T., Sudhakaran R., Okugawa S., Inada M., Kono T., Sakai M., Itami T. (2010). A novel gene of tumor necrosis factor ligand superfamily from kuruma shrimp, *Marsupenaeus japonicus*. Fish Shellfish Immunol..

[66] Gu H.-J., Sun Q.-L., Jiang S., Zhang J., Sun L. (2018). First characterization of an anti-lipopolysaccharide factor (ALF) from hydrothermal vent shrimp: Insights into the immune function of deep-sea crustacean ALF. Dev. Comp. Immunol..

[67] Srinivasan V., Maestroni G.J.M., Cardinali D.P., Esquifino A.I., Perumal S.R.P., Miller S.C. (2005). Melatonin, immune function and aging. Immun. Ageing.

[68] Carrillo-Vico A., Lardone P.J., Alvarez-Sánchez N., Rodríguez-Rodríguez A., Guerrero J.M. (2013). Melatonin: Buffering the immune system. Int. J. Mol. Sci..

[69] Moslehi M., Moazamiyanfar R., Dakkali M.S., Rezaei S., Rastegar-Pouyani N., Jafarzadeh E., Mouludi K., Khodamoradi E., Taeb S., Najafi M. (2022). Modulation of the immune system by melatonin; implications for cancer therapy. Int. Immunopharmacol..

[70] Voreades N., Kozil A., Weir T.L. (2014). Diet and the development of the human intestinal microbiome. Front. Microbiol..

[71] Duan Y., Wang Y., Ding X., Xiong D., Zhang J. (2020). Response of intestine microbiota, digestion, and immunity in Pacific white shrimp *Litopenaeus vannamei* to dietary succinate. Aquaculture.

[72] Hirano T., Yokoyama M., Ikejima M., Shiraishi H., Hakamata W., Nishio T. (2024). Impact of chitin-derived β-N-acetyl-d-glucosaminyl-(1,4)-d-glucosamine on chitinase upregulation in *Shewanella baltica*. FEMS Microbiol. Lett..

[73] Han L., Ren J., Xue Y., Gao J., Fu Q., Shao P., Zhu H., Zhang M., Ding F. (2024). Fatty acid synthesis promoted by PA1895-1897 operon delays quorum sensing activation in *Pseudomonas aeruginosa*. AMB Express.

[74] Zhou Z., Wen M., Xiang L., Shen H., Jiang G., Cheng J., Hu Y., Qian J. (2024). Segmental variations in intestinal microbiota composition and functional capacity along the digestive tract of *Litopenaeus vannamei*. Aquac. Rep..

[75] Zhu H., Qiang J., He J., Tao Y., Bao J., Xu P. (2021). Physiological parameters and gut microbiome associated with different dietary lipid levels in hybrid yellow catfish (*Tachysurus fulvidraco*♀ × *Pseudobagrus vachellii*♂). Comp. Biochem. Physiol. Part D Genom. Proteom..

[76] Turnbaugh P.J., Hamady M., Yatsunenko T., Cantarel B.L., Duncan A., Ley R.E., Sogin M.L., Jones W.J., Roe B.A., Affourtit J.P. (2009). A core gut microbiome in obese and lean twins. Nature.

[77] Baekelandt S., Cornet V., Mandiki S.N.M., Lambert J., Dubois M., Kestemont P. (2021). Ex vivo approach supports both direct and indirect actions of melatonin on immunity in pike-perch *Sander lucioperca*. Fish Shellfish Immunol..

